# Supplementary material for: The kinetochore prevents centromere-proximal crossover recombination during meiosis
Source: eLife. 2015 Dec 14;4:e10850. doi: 10.7554/eLife.10850 (PMC4749563; doi:10.7554/eLife.10850)
Supplement: Supplementary file 4. — (A) List of yeast strains. (B) List of qPCR primers. DOI: http://dx.doi.org/10.7554/eLife.10850.022 [file elife-10850-supp4.docx]

**Supplementary File 4**

Contains:

Supplementary File A: Yeast strains used in this study.

Supplementary File B: qPCR primers used in this study.

**Supplementary File A** Yeast strains

| **Strain** | | **Genotype** | **Figure** |
| --- | --- | --- | --- |
| **AM1835** | | *MATa/MATα* | Figure 1-figure supplement 1C |
| AM4015 | | *MATa/MATα*  *REC8-3HA::URA3/REC8-3HA::URA3*  *ndt80Δ::LEU2/ndt80Δ::LEU2* | Figure 5A-E |
| AM4016 | | *MATa/MATα*  *REC8-3HA::URA3/REC8-3HA::URA3*  *ndt80Δ::LEU2/ndt80Δ::LEU2*  *iml3Δ::KanMX6/iml3Δ::KanMX6* | Figure 5A,B,D |
| AM4017 | | *MATa/MATα*  *REC8-3HA::URA3/REC8-3HA::URA3*  *ndt80Δ::LEU2/ndt80Δ::LEU2*  *chl4Δ::KanMX6/chl4Δ::KanMX6* | Figure 5D |
| AM8769 | | *MATa/MATα*  *ndt80::pGAL-NDT80::TRP1/ndt80::pGAL-NDT80::TRP1*  *ura3::pGPD1-GAL4(848).ER::URA3/ ura3::pGPD1-GAL4(848).ER::URA3*  *NDC10-6HA::HIS3/NDC10-6HA::HIS3* | Figure 7C |
| AM8770 | | *MATa/MATα*  *ndt80::pGAL-NDT80::TRP1/ndt80::pGAL-NDT80::TRP1*  *ura3::pGPD1-GAL4(848).ER::URA3/ ura3::pGPD1-GAL4(848).ER::URA3*  *NDC10-6HA::HIS3/NDC10-6HA::HIS3*  *chl4Δ::KanMX6/chl4Δ::KanMX6* | Figure 7C |
| AM8772 | | *MATa/MATα*  *ndt80::pGAL-NDT80::TRP1/ndt80::pGAL-NDT80::TRP1*  *ura3::pGPD1-GAL4(848).ER::URA3/ ura3::pGPD1-GAL4(848).ER::URA3*  *NDC10-6HA::HIS3/NDC10-6HA::HIS3*  *iml3Δ::KanMX6/iml3Δ::KanMX6* | Figure 7C |
| AM8861 | | *MATa/MATα*  *ndt80::pGAL-NDT80::TRP1/ndt80::pGAL-NDT80::TRP1*  *ura3::pGPD1-GAL4(848).ER::URA3/ ura3::pGPD1-GAL4(848).ER::URA3*  *NDC10-6HA::HIS3/NDC10-6HA::HIS3*  *mcm21Δ::KanMX6/mcm21Δ::KanMX6* | Figure 7C |
| AM9017 | | *MATa/MATα*  *ndt80::pGAL-NDT80::TRP1/ndt80::pGAL-NDT80::TRP1*  *ura3::pGPD1-GAL4(848).ER::URA3/ ura3::pGPD1-GAL4(848).ER::URA3*  *NDC10-6HA::HIS3/NDC10-6HA::HIS3*  *spo11Δ::URA3/spo11Δ::URA3*  *ctf19Δ::KanMX6/ctf19Δ::KanMX6* | Figure 8A |
| AM9018 | | *MATa/MATα*  *ndt80::pGAL-NDT80::TRP1/ndt80::pGAL-NDT80::TRP1*  *ura3::pGPD1-GAL4(848).ER::URA3/ ura3::pGPD1-GAL4(848).ER::URA3*  *NDC10-6HA::HIS3/NDC10-6HA::HIS3*  *spo11Δ::URA3/spo11Δ::URA3* | Figure 8A, B |
| AM9049 | | *MATa/MATα*  *ndt80::pGAL-NDT80::TRP1/ndt80::pGAL-NDT80::TRP1*  *ura3::pGPD1-GAL4(848).ER::URA3/ ura3::pGPD1-GAL4(848).ER::URA3*  *NDC10-6HA::HIS3/NDC10-6HA::HIS3*  *ctf19Δ::KanMX6/ctf19Δ::KanMX6* | Figure 7C |
| AM9287 | | *MATa/MATα*  *ndt80::pGAL-NDT80::TRP1/ndt80::pGAL-NDT80::TRP1*  *ura3::pGPD1-GAL4(848).ER::URA3/ ura3::pGPD1-GAL4(848).ER::URA3*  *NDC10-6HA::HIS3/NDC10-6HA::HIS3*  *spo11Δ::URA3/spo11Δ::URA3*  *chl4Δ::KanMX6/chl4Δ::KanMX6* | Figure 8A |
| AM9288 | | *MATa/MATα*  *ndt80::pGAL-NDT80::TRP1/ndt80::pGAL-NDT80::TRP1*  *ura3::pGPD1-GAL4(848).ER::URA3/ ura3::pGPD1-GAL4(848).ER::URA3*  *NDC10-6HA::HIS3/NDC10-6HA::HIS3*  *spo11Δ::URA3/spo11Δ::URA3*  *iml3Δ::KanMX6/iml3Δ::KanMX6* | Figure 8A, B |
| AM10658 | | *MATa/MATα*  *ndt80Δ::LEU2/ndt80Δ::LEU2*  *chl4Δ::KanMX6/chl4Δ::KanMX6* | Figure 7B |
| AM10660 | | *MATa/MATα*  *ndt80Δ::LEU2/ndt80Δ::LEU2*  *ctf19Δ::KanMX6/ctf19Δ::KanMX6* | Figure 7B |
| AM10664 | | *MATa/MATα*  *ndt80Δ::LEU2/ndt80Δ::LEU2*  *mcm21Δ::KanMX6/mcm21Δ::KanMX6* | Figure 7B,D,F |
| AM10686 | | *MATa/MATα*  *ndt80Δ::LEU2/ndt80Δ::LEU2*  *iml3Δ::KanMX6/iml3Δ::KanMX6* | Figure 7B,D,E |
| AM10913 | | *MATa/MATα*  *ndt80Δ::LEU2/ndt80Δ::LEU2*  *zip1Δ::HIS3/zip1Δ::HIS3* | Figure 7A,B |
| AM11633 | | *MATa/MATα*  *ndt80Δ::LEU2/ndt80Δ::LEU2* | Figure 7A, B, D-F,  Figure 5D,E |
| AM12466 | | *MATa/MATα*  *ndt80Δ::LEU2/ndt80Δ::LEU2*  *leu2::pURA3-TetR-GFP::LEU2/leu2::pURA3-TetR-GFP::LEU2*  *CEN5::tetOx224::HIS3/CEN5::tetOx224::HIS3*  *chl4Δ::KanMX6/chl4Δ::KanMX6* | Figure 8C |
| AM12469 | | *MATa/MATα*  *ndt80Δ::LEU2/ndt80Δ::LEU2*  *leu2::pURA3-TetR-GFP::LEU2/leu2::pURA3-TetR-GFP::LEU2*  *lys2::tetOx240::URA3/lys2::tetOx240::URA3* | Figure 8D |
| AM12823 | | *MATa/MATα*  *ndt80Δ::LEU2/ndt80Δ::LEU2*  *leu2::pURA3-TetR-GFP::LEU2/leu2::pURA3-TetR-GFP::LEU2*  *CEN5::tetOx224::HIS3/+*  *lys2::tetOx240::URA3/+* | Figure 8C,D |
| AM12825 | | *MATa/MATα*  *ndt80Δ::LEU2/ndt80Δ::LEU2*  *leu2::pURA3-TetR-GFP::LEU2/leu2::pURA3-TetR-GFP::LEU2*  *lys2::tetOx240::URA3/lys2::tetOx240::URA3*  *mcm21Δ::KanMX6/mcm21Δ::KanMX6* | Figure 8D |
| AM12829 | | *MATa/MATα*  *ndt80Δ::LEU2/ndt80Δ::LEU2*  *leu2::pURA3-TetR-GFP::LEU2/leu2::pURA3-TetR-GFP::LEU2*  *CEN5::tetOx224::HIS3/CEN5::tetOx224::HIS3* | Figure 8C |
| AM12831 | | *MATa/MATα*  *ndt80Δ::LEU2/ndt80Δ::LEU2*  *leu2::pURA3-TetR-GFP::LEU2/leu2::pURA3-TetR-GFP::LEU2*  *lys2::tetOx240::URA3/lys2::tetOx240::URA3*  *ctf19Δ::KanMX6/ctf19Δ::KanMX6* | Figure 8D |
| AM12837 | | *MATa/MATα*  *ndt80Δ::LEU2/ndt80Δ::LEU2*  *leu2::pURA3-TetR-GFP::LEU2/leu2::pURA3-TetR-GFP::LEU2*  *CEN5::tetOx224::HIS3/CEN5::tetOx224::HIS3*  *mcm21Δ::KanMX6/mcm21Δ::KanMX6* | Figure 8C |
| AM12978 | | *MATa/MATα*  *ndt80Δ::LEU2/ndt80Δ::LEU2*  *leu2::pURA3-TetR-GFP::LEU2/leu2::pURA3-TetR-GFP::LEU2*  *lys2::tetOx240::URA3/lys2::tetOx240::URA3*  *iml3Δ::KanMX6/iml3Δ::KanMX6* | Figure 8D |
| AM12980 | | *MATa/MATα*  *ndt80Δ::LEU2/ndt80Δ::LEU2*  *leu2::pURA3-TetR-GFP::LEU2/leu2::pURA3-TetR-GFP::LEU2*  *lys2::tetOx240::URA3/lys2::tetOx240::URA3*  *chl4Δ::KanMX6/chl4Δ::KanMX6* | Figure 8D |
| AM13149 | | *MATa/MATα*  *THR1::pYKL050c-CFP::TRP/THR1::pYKL050c-CFP::TRP*  *CEN8::pYKL050c-RFP::LEU/+*  *SGD.115024-115572::pYKL050c-GFP*::URA/+* | Figure 1B,  Figure 5F,  Figure 8E |
| AM13346 | | *MATa/MATα*  *ndt80Δ::LEU2/ndt80Δ::LEU2*  *leu2::pURA3-TetR-GFP::LEU2/leu2::pURA3-TetR-GFP::LEU2*  *CEN5::tetOx224::HIS3/CEN5::tetOx224::HIS3*  *ctf19Δ::KanMX6/ctf19Δ::KanMX6* | Figure 8C |
| AM13348 | | *MATa/MATα*  *ndt80Δ::LEU2/ndt80Δ::LEU2*  *leu2::pURA3-TetR-GFP::LEU2/leu2::pURA3-TetR-GFP::LEU2*  *CEN5::tetOx224::HIS3/CEN5::tetOx224::HIS3*  *iml3Δ::KanMX6/iml3Δ::KanMX6* | Figure 8C |
| AM13408 | | *MATa/MATα*  *THR1::pYKL050c-CFP::TRP/THR1::pYKL050c-CFP::TRP*  *CEN8::pYKL050c-RFP::LEU/+*  *SGD.115024-115572::pYKL050c-GFP*::URA/+*  *iml3Δ::KanMX6/iml3Δ::KanMX6* | Figure 1B |
| AM13410 | | *MATa/MATα*  *THR1::pYKL050c-CFP::TRP/THR1::pYKL050c-CFP::TRP*  *CEN8::pYKL050c-RFP::LEU/+*  *SGD.115024-115572::pYKL050c-GFP*::URA/+*  *chl4Δ::KanMX6/chl4Δ::KanMX6* | Figure 1B |
| AM13411 | | *MATa/MATα*  *THR1::pYKL050c-CFP::TRP/THR1::pYKL050c-CFP::TRP*  *CEN8::pYKL050c-RFP::LEU/+*  *SGD.115024-115572::pYKL050c-GFP*::URA/+*  *ctf19Δ::KanMX6/ctf19Δ::KanMX6* | Figure 1B |
| AM13413 | | *MATa/MATα*  *THR1::pYKL050c-CFP::TRP/THR1::pYKL050c-CFP::TRP*  *CEN8::pYKL050c-RFP::LEU/+*  *SGD.115024-115572::pYKL050c-GFP*::URA/+*  *mcm21Δ::KanMX6/mcm21Δ::KanMX6* | Figure 1B,  Figure 3F |
| AM13776 | | *MATa/MATα*  *ndt80::pGAL-NDT80::TRP1/ndt80::pGAL-NDT80::TRP1*  *ura3::pGPD1-GAL4(848).ER::URA3/ ura3::pGPD1-GAL4(848).ER::URA3*  *NDC10-6HA::HIS3/NDC10-6HA::HIS3*  *spo11Δ::URA3/spo11Δ::URA3*  *mcm21Δ::KanMX6/mcm21Δ::KanMX6* | Figure 8A, B |
| AM13833 | | *MATa/MATα*  *REC8-3HA::URA3/REC8-3HA::URA3*  *ndt80Δ::LEU2/ndt80Δ::LEU2*  *mcm21Δ::KanMX6/mcm21Δ::KanMX6* | Figure 5A,C,D |
| AM13964 | | *MATa/MATα*  *THR1::pYKL050c-CFP::TRP/THR1::pYKL050c-CFP::TRP*  *CEN8::pYKL050c-RFP::LEU/+*  *SGD.115024-115572::pYKL050c-GFP*::URA/+*  *zip1Δ::HIS3/zip1Δ::HIS3* | Figure 1B,  Figure 8E |
| AM14087 | | *MATa/MATα*  *THR1::pYKL050c-CFP::TRP/THR1::pYKL050c-CFP::TRP*  *ARG4::pYKL050c-GFP*::URA/+*  *SGD.150521-151070::pYKL050c-RFP::LEU/+* | Figure 1C  Figure 5G, Figure 8F |
| AM14089 | | *MATa/MATα*  *THR1::pYKL050c-CFP::TRP/THR1::pYKL050c-CFP::TRP*  *CEN8::pYKL050c-RFP::LEU/+*  *SGD.115024-115572::pYKL050c-GFP*::URA/+*  *zip1Δ::URA3/zip1Δ::URA3*  *trp1::zip1-S75E::TRP1/trp1::zip1-S75E::TRP1* | Figure 8E |
| AM14235 | | *MATa/MATα*  *THR1::pYKL050c-CFP::TRP/THR1::pYKL050c-CFP::TRP*  *ARG4::pYKL050c-GFP*::URA/+*  *SGD.150521-151070::pYKL050c-RFP::LEU/+*  *mcm21Δ::KanMX6/mcm21Δ::KanMX6* | Figure 1C |
| AM14237 | | *MATa/MATα*  *THR1::pYKL050c-CFP::TRP/THR1::pYKL050c-CFP::TRP*  *ARG4::pYKL050c-GFP*::URA/+*  *SGD.150521-151070::pYKL050c-RFP::LEU/+*  *chl4Δ::KanMX6/chl4Δ::KanMX6* | Figure 1C |
| AM14239 | | *MATa/MATα*  *THR1::pYKL050c-CFP::TRP/THR1::pYKL050c-CFP::TRP*  *ARG4::pYKL050c-GFP*::URA/+*  *SGD.150521-151070::pYKL050c-RFP::LEU/+*  *ctf19Δ::KanMX6/ctf19Δ::KanMX6* | Figure 1C |
| AM14243 | | *MATa/MATα*  *THR1::pYKL050c-CFP::TRP/THR1::pYKL050c-CFP::TRP*  *ARG4::pYKL050c-GFP*::URA/+*  *SGD.150521-151070::pYKL050c-RFP::LEU/+*  *iml3Δ::KanMX6/iml3Δ::KanMX6* | Figure 1C |
| AM14247 | | *MATa/MATα*  *THR1::pYKL050c-CFP::TRP/THR1::pYKL050c-CFP::TRP*  *ARG4::pYKL050c-GFP*::URA/+*  *SGD.150521-151070::pYKL050c-RFP::LEU/+*  *zip1Δ::HIS3/zip1Δ::HIS3* | Figure 1C,  Figure 8F |
| AM14289 | | *MATa/MATα*  *THR1::pYKL050c-CFP::TRP/THR1::pYKL050c-CFP::TRP*  *ARG4::pYKL050c-GFP*::URA/+*  *SGD.150521-151070::pYKL050c-RFP::LEU/+*  *zip1Δ::URA3/zip1Δ::URA3*  *trp1::zip1-S75E::TRP1/trp1::zip1-S75E::TRP1* | Figure 8F |
| AM15182  (YJM789 background) | | *MATα*  *iml3::pCLB2-3HA-IML3::KanMX6/iml3::pCLB2-3HA-IML3::KanMX6* | Figure 2C-F,  Figure 2-figure supplement 2A-C |
| AM15183  (S96 background) | | *MATa*  *iml3::pCLB2-3HA-IML3::KanMX6/iml3::pCLB2-3HA-IML3::KanMX6* | Figure 2C-F,  Figure 2-figure supplement 2A-C |
| AM16217 | | *MATa/MATα*  *THR1::pYKL050c-CFP::TRP/THR1::pYKL050c-CFP::TRP*  *ARG4::pYKL050c-GFP*::URA/+*  *SGD.150521-151070::pYKL050c-RFP::LEU/+*  *scc4-m35::HIS3/scc4-m35::HIS3* | Figure 5G |
| AM16446  (SKY5100) | | *MATa/MATα*  *SPO11-6His-3FLAG-loxP-KanMX6-loxP/ SPO11-6His-3FLAG-loxP-KanMX6-loxP*  *ho::LYS2/ho::LYS2*  *ura3/ura3, lys2/lys2, leu2/leu2, arg4-Bgl/arg4-Bgl*  *nuc1Δ::LEU2/nuc1Δ::LEU2*  *mcm21Δ::NAT/mcm21Δ::NAT* | Figure 3B-E  Figure 3-figure supplement 1 |
| AM16798 | | *MATa/MATα*  *THR1::pYKL050c-CFP::TRP/THR1::pYKL050c-CFP::TRP*  *ARG4::pYKL050c-GFP*::URA/+*  *SGD.150521-151070::pYKL050c-RFP::LEU/+*  *SCC4::HIS3/SCC4::HIS3* | Figure 5G |
| AM16864 | | *MATa/MATα*  *THR1::pYKL050c-CFP::TRP/THR1::pYKL050c-CFP::TRP*  *CEN8::pYKL050c-RFP::LEU/+*  *SGD.115024-115572::pYKL050c-GFP*::URA/+*  *scc4-m35::HIS3/scc4-m35::HIS3* | Figure 5F |
| AM17381 | | *MATa/MATα*  *THR1::pYKL050c-CFP::TRP/THR1::pYKL050c-CFP::TRP*  *CEN8::pYKL050c-RFP::LEU/+*  *SGD.115024-115572::pYKL050c-GFP*::URA/+*  *SCC4::HIS3/SCC4::HIS3* | Figure 5F |
| AM17552 | | *MATa/MATα*  *THR1::pYKL050c-CFP::TRP/THR1::pYKL050c-CFP::TRP*  *CEN8::pYKL050c-RFP::LEU/+*  *SGD.115024-115572::pYKL050c-GFP*::URA/+*  *iml3::pCLB2-3HA-IML3::KanMX6/iml3::pCLB2-3HA-IML3::KanMX6* | Figure 1B, Figure 2-figure supplement 1 |
| AM17554 | | *MATa/MATα*  *THR1::pYKL050c-CFP::TRP/THR1::pYKL050c-CFP::TRP*  *ARG4::pYKL050c-GFP*::URA/+*  *SGD.150521-151070::pYKL050c-RFP::LEU/+*  *iml3::pCLB2-3HA-IML3::KanMX6/iml3::pCLB2-3HA-IML3::KanMX6* | Figure 1C |
| AM17700 | | *MATa/MATα*  *THR1::pYKL050c-CFP::TRP/THR1::pYKL050c-CFP::TRP*  *CEN8::pYKL050c-RFP::LEU/+*  *SGD.115024-115572::pYKL050c-GFP*::URA/+*  *nkp1Δ::KanMX6/nkp1Δ::KanMX6* | Figure 1B |
| AM17702 | | *MATa/MATα*  *THR1::pYKL050c-CFP::TRP/THR1::pYKL050c-CFP::TRP*  *CEN8::pYKL050c-RFP::LEU/+*  *SGD.115024-115572::pYKL050c-GFP*::URA/+*  *cnn1Δ::KanMX6/cnn1Δ::KanMX6* | Figure 1B |
| AM17704 | | *MATa/MATα*  *THR1::pYKL050c-CFP::TRP/THR1::pYKL050c-CFP::TRP*  *CEN8::pYKL050c-RFP::LEU/+*  *SGD.115024-115572::pYKL050c-GFP*::URA/+*  *nkp2Δ::KanMX6/nkp2Δ::KanMX6* | Figure 1B |
| AM17803 | | *MATa/MATα*  *THR1::pYKL050c-CFP::TRP/THR1::pYKL050c-CFP::TRP*  *CEN8::pYKL050c-RFP::LEU/+*  *SGD.115024-115572::pYKL050c-GFP*::URA/+*  *mhf1Δ::NAT/mhf1Δ::NAT* | Figure 1B |
| AM17805 | | *MATa/MATα*  *THR1::pYKL050c-CFP::TRP/THR1::pYKL050c-CFP::TRP*  *CEN8::pYKL050c-RFP::LEU/+*  *SGD.115024-115572::pYKL050c-GFP*::URA/+*  *mhf2Δ::NAT/mhf2Δ::NAT* | Figure 1B |
| AM17807 | | *MATa/MATα*  *THR1::pYKL050c-CFP::TRP/THR1::pYKL050c-CFP::TRP*  *CEN8::pYKL050c-RFP::LEU/+*  *SGD.115024-115572::pYKL050c-GFP*::URA/+*  *mhf1Δ::NAT/mhf1Δ::NAT* | Figure 1B |
| AM17809 | | *MATa/MATα*  *THR1::pYKL050c-CFP::TRP/THR1::pYKL050c-CFP::TRP*  *CEN8::pYKL050c-RFP::LEU/+*  *SGD.115024-115572::pYKL050c-GFP*::URA/+*  *wip1Δ::NAT/wip1Δ::NAT* | Figure 1B |
| AM18054 | | *MATa/MATα*  *THR1::pYKL050c-CFP::TRP/THR1::pYKL050c-CFP::TRP*  *ARG4::pYKL050c-GFP*::URA/+*  *SGD.150521-151070::pYKL050c-RFP::LEU/+*  *cnn1Δ::KanMX6/cnn1Δ::KanMX6* | Figure 1C |
| AM18098 | | *MATa/MATα*  *THR1::pYKL050c-CFP::TRP/THR1::pYKL050c-CFP::TRP*  *ARG4::pYKL050c-GFP*::URA/+*  *SGD.150521-151070::pYKL050c-RFP::LEU/+*  *nkp1Δ::KanMX6/nkp1Δ::KanMX6* | Figure 1C |
| AM18211 | | *MATa/MATα*  *REC8-3HA::URA3/REC8-3HA::URA3*  *ndt80Δ::LEU2/ndt80Δ::LEU2*  *scc4-m35::HIS3/scc4-m35::HIS3* | Figure 5E |
| AM18881 | | *MATa/MATα*  *ndt80Δ::LEU2/ndt80Δ::LEU2*  *scc4-m35::HIS3/scc4-m35::HIS3* | Figure 7A |
| AM18978 | | *MATa/MATα*  *tor1-1::HIS3/ tor1-1::HIS3 fpr1::KanMX4/fpr1::KanMX4 ndt80Δ::NATMX6/ndt80Δ::NATMX6*  *RPL13A-2XFKBP12::TRP1/ RPL13A-2XFKBP12::TRP1*  *REC8-3HA::URA3/REC8-3HA::URA3*  *CTF19-FRB-GFP::KanMX6/ CTF19-FRB-GFP::KanMX6* | Figure 6D |
| AM19543 | | *MATa/MATalpha*  *THR1::pYKL050c-CFP::TRP/THR1::pYKL050c-CFP::TRP*  *CEN8::pYKL050c-RFP::LEU/+*  *SGD.115024-115572::pYKL050c-GFP*::URA/+*  *ndt80::pGAL-NDT80::TRP1/ndt80::pGAL-NDT80::TRP1*  *ura3::pGPD1-GAL4(848).ER::URA3/ ura3::pGPD1-GAL4(848).ER::URA3*  *RPL13A-2XFKBP12::TRP1/ RPL13A-2XFKBP12::TRP1*  *tor1-1::HIS3/ tor1-1::HIS3 fpr1::KanMX4/fpr1::KanMX4*  *CTF19-FRB-GFP::KanMX6/ CTF19-FRB-GFP::KanMX6* | Figure 6K |
| AM20078 | *MATa/MATα*  *ndt80Δ::LEU2/ndt80Δ::LEU2*  *DSN1-6HIS-3FLAG::URA3/ DSN1-6HIS-3FLAG::URA3* | | Figure 1-figure supplement 1 |
| AM20080 | *MATa/MATα*  *ndt80Δ::LEU2/ndt80Δ::LEU2*  *DSN1-6HIS-3FLAG::URA3/ DSN1-6HIS-3FLAG::URA3*  *mcm21Δ::KanMX6/mcm21Δ::KanMX6* | | Figure 1-figure supplement 1 |
| AM20082 | *MATa/MATα*  *dsn1::pCLB2-3HA-DSN1::KanMX6/ dsn1::pCLB2-3HA-DSN1::KanMX6* | | Figure 1-figure supplement 1 |
| AM20084 | *MATa/MATα*  *mtw1::pCLB2-3HA-MTW1::KanMX6/mtw1::pCLB2-3HA-MTW1::KanMX6* | | Figure 1-figure supplement 1 |
| AM20086 | *MATa/MATα*  *REC8-3HA::URA3/REC8-3HA::URA3*  *ndt80Δ::LEU2/ndt80Δ::LEU2*  *ctf19Δ::KanMX6/ctf19Δ::KanMX6* | | Figure 5D |
| AM20294 | *MATa/MATα*  *mtw1::pCLB2-3HA-MTW1::KanMX6/mtw1::pCLB2-3HA-MTW1::KanMX6*  *ndt80Δ::LEU2/ndt80Δ::LEU2*  *MCM21-yEGFP::KanMX6/MCM21-yEGFP::KanMX6* | | Figure 1-figure supplement 1B |
| AM20295 | *MATa/MATα*  *dsn1::pCLB2-3HA-DSN1::KanMX6/dsn1::pCLB2-3HA-DSN1::KanMX6*  *REC8-3HA::URA3/-*  *ndt80Δ::LEU2/ndt80Δ::LEU2*  *MCM21-yEGFP::KanMX6/MCM21-yEGFP::KanMX6* | | Figure 1-figure supplement 1B |
| AM20296 | *MATa/MATα*  *ndt80Δ::LEU2/ndt80Δ::LEU2*  *MCM21-yEGFP::KanMX6/MCM21-yEGFP::KanMX6* | | Figure 1-figure supplement 1B |
|  |  | |  |
| AM20138 | *MATa/MATα*  *ndt80::pGAL-NDT80::TRP1/ndt80::pGAL-NDT80::TRP1*  *ura3::pGPD1-GAL4(848).ER::URA3/ura3::pGPD1-GAL4(848).ER::URA3*  *RPL13A-2XFKBP12::TRP1/RPL13A-2XFKBP12::TRP1*  *tor1-1::HIS3/tor1-1::HIS3*  *fpr1::KanMX4/fpr1::KanMX4*  *CTF19-FRB-GFP::KanMX6/CTF19-FRB-GFP::KanMX6*  *NDC10-6HA::HIS3/NDC10-6HA::HIS3*  *REC8-13MYC::KanMX6/REC8-13MYC::KanMX6* | | Figure 6E,F |
| GV48 | | *MATa, ho::LYS2, lys2, leu2::hisG, his4X::LEU2-URA3, ura3,* *arg4-nsp, dmc1Δ::ARG4*  *MATα, ho::LYS2, lys2, leu2::hisG, his4B::LEU2, ura3, arg4-Bgl2, dmc1Δ::ARG4* | Figure 4B,  Figure 4-figure supplement 1,  Figure 6G, I,J, |
| GV1853 | | *MATa, ho::LYS2, lys2, ura3, leu2::hisG, his3::hisG, trp1::hisG, RPL13A-2XFKBP12::TRP1, fpr1::KanMX4, tor1-1::HIS3*  *MATα, ho::LYS2, lys2, ura3, leu2::hisG, his3::hisG, trp1::hisG, RPL13A-2XFKBP12::TRP1, fpr1::KanMX4, tor1-1::HIS3* | Figure 6-figure supplement 1A,B |
| GV1870 | | *MATa, ho::LYS2, lys2, leu2::hisG, his4X::LEU2-URA3, ura3, arg4-nsp, dmc1Δ::ARG4, TRP1, iml3Δ::KANMX*  *MATα, ho::LYS2, lys2, leu2::hisG, ura3, arg4-nsp, dmc1Δ::ARG4, TRP1, ARG4, iml3Δ::KANMX* | Figure 4C, Figure 4-figure supplement 1 |
| GV1906 | | *MATa, ho::LYS2, lys2, leu2::hisG, his4X::LEU2-URA3, ura3, ARG4,*  *TRP1, dmc1Δ::ARG4, nkp2ΔKanMX6*  *MATα, ho::LYS2, lys2, leu2::hisG, his4X::LEU2-URA3, ura3, ARG4, trp1::hisG, dmc1Δ::ARG4, nkp2ΔKanMX6* | Figure 4C |
| GV1908 | | *MATa, ho::LYS2, lys2, leu2::hisG, his4X::LEU2-URA3, ura3, ARG4 (?),TRP1, dmc1Δ::ARG4, nkp1ΔKanMX6*  *MATα, ho::LYS2, lys2, leu2::hisG, his4X::LEU2-URA3, ura3, arg4(-nsp),TRP1, dmc1Δ::ARG4, nkp1ΔKanMX6* | Figure 4C |
| GV1912 | | *MATa, ho::LYS2, lys2, leu2::hisG, his4X::LEU2-URA3, ura3, ARG4/ arg4(-nsp), TRP1, dmc1Δ::ARG4, ctf19ΔKanMX6*  *MATα, ho::LYS2, lys2, leu2::hisG, his4X::LEU2-URA3, ura3, arg4(-nsp), TRP1, dmc1Δ::ARG4, ctf19ΔKanMX6* | Figure 4B,C  Figure 4-figure supplement 1  Figure 6I, J, I |
| GV1913 | | *MATa, ho::LYS2, lys2, leu2::hisG, his4X::LEU2-URA3, ura3, arg4(-nsp),TRP1, dmc1Δ::ARG4, ctf3ΔKanMX6*  *MATα, ho::LYS2, lys2, leu2::hisG, his4X::LEU2-URA3, ura3, TRP1, dmc1Δ::ARG4, ARG4, ctf3ΔKanMX6* | Figure 4C |
| GV1914 | | *MATa, ho::LYS2, lys2, leu2::hisG, his4X::LEU2-URA3, ARG4 (?), ura3, TRP1, dmc1Δ::ARG4, mcm16Δ::KanMX6*  *MATα, ho::LYS2, lys2, leu2::hisG, his4X::LEU2-URA3, ARG4, ura3, trp1::hisG, dmc1Δ::ARG4, mcm16Δ::KanMX6* | Figure 4C |
| GV1971 | | *MATa, ho::LYS2, lys2, ura3, leu2::hisG, his3::hisG, trp1::hisG, arg4-Bgl2/ARG4, mcm22Δ::KanMX, dmc1Δ::ARG4*  *MATα, ho::LYS2, lys2, ura3, leu2::hisG, his3::hisG, trp1::hisG, his4B::LEU2, , arg4-Bgl2/ARG4, mcm22Δ::KanMX, dmc1Δ::ARG4* | Figure 4C |
| GV2029 | | *MATa, ho::LYS2, lys2, leu2::hisG, his4B::LEU2, ura3, ARG4, TRP, HIS, dmc1Δ::ARG4, cnn1Δ::HphMX*  *MATα, ho::LYS2, lys2, leu2::hisG, his4B::LEU2, ura3, ARG4, TRP, dmc1Δ::ARG4, cnn1Δ::HphMX* | Figure 4C |
| GV2050 | | *MATa, ho::LYS2, lys2, leu2::hisG, ura3, arg4-Bgl2/ARG4, dmc1Δ::ARG4, mcm21Δ::KANMX*  *MATα, ho::LYS2, lys2, his4B::LEU2, ura3, arg4-Bgl2/ARG4, dmc1Δ::ARG4, mcm21Δ::KANMX* | Figure 4B,C  Figure 6J |
| GV2128 | | *MATa, ho::LYS2, lys2, leu2::hisG, ura3, ARG4 (?), TRP1, dmc1Δ::ARG4, ctf19ΔKanMX6, spo11-Y135F-HA-URA3*  *MATα, ho::LYS2, lys2, leu2::hisG, ura3, ARG4 (?), TRP1, dmc1Δ::ARG4, ctf19ΔKanMX6, spo11-Y135F-HA-URA3* | Figure 4B |
| GV2139 | | *MATa, ho::LYS2, lys2, leu2::hisG, his4X::LEU2-URA3, ura3, arg4-nsp, dmc1Δ::ARG4, TRP1, ARG4, chl4Δ::KANMX*  *MATα, ho::LYS2, lys2, leu2::hisG, trp1::hisG, ura3, arg4-nsp, dmc1Δ::ARG4, TRP1, ARG4, chl4Δ::KANMX* | Figure 4C,  Figure 4-figure supplement 1, |
| GV2205 | | *MATa, ho::LYS2, lys2, ura3::hisG, leu2::hisG, his4X, TRP1, spo11-Y135F-HA-URA3, arg4-Bgl2/ARG4, dmc1Δ::ARG4, mcm21Δ::KANMX*  *MATα, ho::LYS2, lys2, ura3::hisG, leu2::hisG, his4X, TRP1, spo11-Y135F-HA-URA3, arg4-Bgl2/ARG4, dmc1Δ::ARG4, mcm21Δ::KANMX* | Figure 4B |
| GV2275 | | *MATa, ho::LYS2, lys2, ura3, leu2::hisG, his3::hisG, trp1::hisG,*  *tor1-1::HIS3, RPL13A-2XFKBP12::TRP1, fpr1::KanMX4, CTF19-FRB-GFP::KanMX6*  *MATα, ho::LYS2, lys2, ura3, leu2::hisG, his3::hisG, trp1::hisG,*  *tor1-1::HIS3, RPL13A-2XFKBP12::TRP1, fpr1::KanMX4, CTF19-FRB-GFP::KanMX6* | Figure 6-figure supplement 1A,B |
| GV2286 | | *MATa, ho::LYS2, lys2, his4B::LEU2, ura3, arg4-Bgl2/ARG4, dmc1Δ::ARG4, mcm21Δ::KANMX, rec8Δ::HIS3MX6*  *MATα, ho::LYS2, lys2, his4B::LEU2, ura3, arg4-Bgl2/ARG4, dmc1Δ::ARG4, mcm21Δ::KANMX, rec8Δ::HIS3MX6* | Figure 6I |
| GV2305 | | *MATa, ho::LYS2, lys2, ura3, leu2::hisG, his3::hisG, trp1::hisG, SCC4::HIS3, his4X::LEU2-URA3, ura3, arg4-nsp, dmc1Δ::ARG4, TRP1, ARG4(?)*  *MATα, ho::LYS2, lys2, ura3, leu2::hisG, his3::hisG, trp1::hisG, SCC4::HIS3, his4X::LEU2-URA3, ura3, arg4-nsp, dmc1Δ::ARG4, TRP1, ARG4* | Figure 6J |
| GV2354 | | *MATa, ho::LYS2, lys2, ura3, leu2::hisG, his3::hisG, trp1::hisG,*  *tor1-1::HIS3, RPL13A-2XFKBP12::TRP1, fpr1::KanMX4, CTF19-FRB-GFP::KanMX6, dmc1Δ::ARG4*  *MATα, ho::LYS2, lys2, ura3, leu2::hisG, his3::hisG, trp1::hisG,*  *tor1-1::HIS3, RPL13A-2XFKBP12::TRP1, fpr1::KanMX4, CTF19-FRB-GFP::KanMX6, dmc1Δ::ARG4* | Figure 6G,H |
| GV2367 | | *MATa, ho::LYS2, lys2, leu2::hisG, ura3, arg4-Bgl2, his3::hisG, trp1::hisG, RPL13A-2XFKBP12::TRP1, fpr1::KanMX4, tor1-1::HIS3, dmc1Δ::ARG4*  *MATα, ho::LYS2, lys2, leu2::hisG, ura3, arg4-Bgl2, his3::hisG, trp1::hisG, RPL13A-2XFKBP12::TRP1, fpr1::KanMX4, tor1-1::HIS3, dmc1Δ::ARG4* | Figure 6G,H |
| GV2403 | | *MATa, ho::LYS2, lys2, ura3, leu2::hisG, his3::hisG, TRP, rec8::HIS3MX6, dmc1Δ::ARG4*  *MATalpha, ho::LYS2, lys2, ura3, leu2::hisG, his3::hisG, TRP1,his4X::LEU2-URA3, his3::hisG, ura3, dmc1Δ::ARG4, pch2Δ::KanMX, rec8::HIS3MX6* | Figure 6I |
| GV2533 | | *MATa, ho::LYS2, lys2, ura3, leu2::hisG, his3::hisG, trp1::hisG, scc4-m35::HIS3, arg4-nsp, dmc1Δ::ARG4, TRP1, ARG4*  *MATalpha, ho::LYS2, lys2, ura3, leu2::hisG, his3::hisG, trp1::hisG, scc4-m35::HIS3, his4X::LEU2-URA3, ura3, arg4-nsp, dmc1Δ::ARG4, TRP1* | Figure 6J |
| GV 2548 | | *MATa, ho::LYS2, lys2, leu2::hisG, ura3, his3::hisG, trp1::hisG, TRP, arg4-nsp/ARG4, REC8-3HA::URA3 (?), dsn1::pCLB2-3HA-DSN1::KanMX6, dmc1Δ::ARG4,*  *MATalpha, ho::LYS2, lys2, leu2::hisG, ura3, his3::hisG, trp1::hisG,  dsn1::pCLB2-3HA-DSN1::KanMX6, arg4-nsp/ARG4, dmc1Δ::ARG4* | Figure 4-figure supplement 1B |
| GV 2734 | | *MATa, ho::LYS2, lys2, leu2::hisG, his4X::LEU2::URA3, ura3, his3::hisG, zip1::LYS2, dmc1Δ::HIS3*  *MATalpha, ho::LYS2, lys2, leu2::hisG, HIS4::LEU2, ura3, HIS3, zip1::LYS2, dmc1Δ::HIS3* | Figure 7- figure supplement 1 |

**Supplementary File B** Primers used for qPCR analysis

| **Locus** | **Primer** | **Oligonucleotide sequence** | **size** | **Position** | **Primer efficiency (SYBR GreenER)** |
| --- | --- | --- | --- | --- | --- |
| Arm1 | AM782 | AGATGAAACTCAGGCTACCA | 93bp | 95kb left of *CEN4* | 2.013 |
|  | AM783 | TGCAACATCGTTAGTTCTTG |  |  |  |
| *CEN4* | AM794 | CCGAGGCTTTCATAGCTTA | 80bp | 150bp right of *CEN4* | 2.061 |
|  | AM795 | ACCGGAAGGAAGAATAAGAA |  |  |  |
| *CEN5* | AM945 | TGAAGGTGAGCTTAAGACAG | 114bp | ~240bp right of *CEN5* | 1.891 |
|  | AM946 | CAACCATGTTCGTAGCTAAA |  |  |  |
| Arm2 | AM975 | TTCTATCGCGTTTAAGTGTG | 89bp | ~215kb right of *CEN5* | 1.927 |
|  | AM976 | TAGTTGATCGTATGGCAGAG |  |  |  |
| *CEN3* | AM1279 | TGTTGATGGGTTTACAATTT | 90bp | ~170bp right of *CEN3* | 1.958 |
|  | AM1280 | CTTTCAATGATTGCTCTAAATC |  |  |  |
| Arm3 | AM1285 | ATGGTACCTAGCTCGTGAAT | 114bp | ~103kb right of *CEN3* | 1.947 |
|  | AM1286 | GGATTTGTCAACTTGGAACT |  |  |  |
| Pericen1 | AM1319 | ATGATTCAATGGATTTAGCC | 103bp | 9.5kb left of *CEN4* | 1.919 |
|  | AM1320 | GTCAGTCTTATGCTGTTCCC |  |  |  |
| Pericen2 | AM1402 | TTCAAACTAATGCGGTAGAG | 85bp | 3kb left of *CEN4* | 1.955 |
|  | AM1403 | TAAATACTACCCATGCACCA |  |  |  |
